# Pneumococcal Capsular Switching: A Historical Perspective

**DOI:** 10.1093/infdis/jis703

**Published:** 2012-11-21

**Authors:** Kelly L. Wyres, Lotte M. Lambertsen, Nicholas J. Croucher, Lesley McGee, Anne von Gottberg, Josefina Liñares, Michael R. Jacobs, Karl G. Kristinsson, Bernard W. Beall, Keith P. Klugman, Julian Parkhill, Regine Hakenbeck, Stephen D. Bentley, Angela B. Brueggemann

**Affiliations:** 1 Department of Zoology, University of Oxford, and; 2 Pathogen Genomics Team, Wellcome Trust Sanger Institute, Hinxton, United Kingdom; 3 Department of Microbiology Surveillance and Research, Statens Serum Institut, Copenhagen, Denmark; 4 Streptococcus Laboratory, Centers for Disease Control and Prevention, and; 5 Hubert Department of Global Health, Emory University, Atlanta, Georgia; 6 Department of Pathology, Case Western Reserve University, Cleveland, Ohio; 7 Centre for Respiratory Diseases and Meningitis, National Institute for Communicable Diseases, Gauteng, South Africa; 8 Department of Microbiology, Bellvitge Hospital-CIBERes-IDIBELL-UB, Barcelona, Spain; 9 Clinical Microbiology Department, Landspitali University Hospital and University of Iceland, Reykjavik;; 10 Department of Microbiology, University Kaiserslautern, Germany

**Keywords:** Capsule, serotype, switching, pneumococcus

## Abstract

***Background.***Changes in serotype prevalence among pneumococcal populations result from both serotype replacement and serotype (capsular) switching. Temporal changes in serotype distributions are well documented, but the contribution of capsular switching to such changes is unknown. Furthermore, it is unclear to what extent vaccine-induced selective pressures drive capsular switching.

***Methods.***Serotype and multilocus sequence typing data for 426 pneumococci dated from 1937 through 2007 were analyzed. Whole-genome sequence data for a subset of isolates were used to investigate capsular switching events.

***Results.***We identified 36 independent capsular switch events, 18 of which were explored in detail with whole-genome sequence data. Recombination fragment lengths were estimated for 11 events and ranged from approximately 19.0 kb to ≥58.2 kb. Two events took place no later than 1960, and the imported DNA included the capsular locus and the nearby penicillin-binding protein genes *pbp2x* and *pbp1a*.

***Conclusions.***Capsular switching has been a regular occurrence among pneumococcal populations throughout the past 7 decades. Recombination of large DNA fragments (>30 kb), sometimes including the capsular locus and penicillin-binding protein genes, predated both vaccine introduction and widespread antibiotic use. This type of recombination has likely been an intrinsic feature throughout the history of pneumococcal evolution.

*Streptococcus pneumoniae* (the “pneumococcus”) is an asymptomatic colonizer of the human nasopharynx and a major cause of otitis media, sinusitis, pneumonia, and meningitis, resulting in approximately 14.5 million annual serious disease episodes globally among young children [1]. The primary virulence determinant is its polysaccharide capsule, of which there are 94 known serotypes [2–6]. The capsule protects against phagocytosis during invasive pneumococcal disease and may also prevent clearance during nasopharyngeal colonization [7, 8]. Ninety-two capsule types are synthesized and exported through the “Wzy-dependent” pathway, whereby extracellular polymerization of component lipid-linked repeat units is preceded by “flippase-mediated” transfer across the cell membrane [2, 9]. The proteins involved are encoded by the capsule polysaccharide synthesis (*cps*) genes, located between *dexB* and *aliA* on the chromosome. The remaining 2 capsule types are synthesized through independent biochemical pathways.

Sequence analyses of 88 reference *cps* loci revealed variable lengths (approximately 10–30 kb) and a range of genes specific to capsule production [2]. The serotype-nonspecific genes were present among all Wzy-dependent capsule types, whereas serotype-specific genes were only found among 1 or a subset of types. Unique serotypes, and further diversity within serogroups, evolved through a combination of mutation and interspecies/intraspecies recombination [2, 10–13].

Serotype is a key determinant of invasive pneumococcal disease potential and prevalence; certain serotypes are more commonly associated with carriage and others more commonly with invasive pneumococcal disease [14, 15]. A 7-valent pneumococcal conjugate vaccine (PCV7) was introduced in the United States in 2000 [16] and, subsequently, into many other countries. Ten-valent and 13-valent vaccines (PHiD-CV and PCV13, respectively) contain polysaccharides targeted against the original 7 serotypes (4, 6B, 9V, 14, 18C, 19F, and 23F) plus serotypes 1, 5, and 7F (both vaccines) and 3, 6A, and 19A (PCV13 only) and have now replaced PCV7 [17, 18].

Surveillance following PCV7 introduction showed a decline of vaccine type (VT) and an increase of nonvaccine type (NVT) pneumococci in disease and nasopharyngeal carriage [19]. This can be attributed to the phenomena of “serotype replacement,” the expansion of preexisting NVT pneumococci, and/or “serotype switching,” a change of serotype of a single clone by alteration or exchange of its *cps* locus [20]. (Serotype switching was first described by Griffith in 1928 [21] and was the focus of the transformation studies by Avery and colleagues in 1944 [22].) These effects are not completely independent: capsular switch variants can subsequently expand within a population. Both phenomena can be studied by comparison of the serotypes and genotypes present in populations before and after introduction of pneumococcal vaccines. Pneumococcal genotypes, as defined by multilocus sequence typing (MLST) [23], show serotype-specific associations [14, 24]; any isolate exemplifying a different genotype/serotype combination may represent a capsular switch variant. Such variants usually arise by recombination at the *cps* locus, and studies of a limited number of such strains indicated that recombination fragment sizes varied from approximately 21.9 kb to approximately 56.5 kb [25–28]. In some cases, the fragments also included part or all of the *pbp2x* and *pbp1a* genes (2 of the 3 primary penicillin-resistance determining genes, located approximately 8 kb upstream and approximately 7 kb downstream of the *cps* locus).

Vaccine-induced selective pressure is contributing to the postvaccination changes of serotype epidemiology, but natural fluctuations in serotype prevalence also play a role. Many studies have documented prevaccine temporal changes in relative serotype prevalence: all but 3 included pneumococci isolated no earlier than 1969; only 2 studies provided genotype data (Supplementary Materials). In this study, we used serotyping, MLST, and whole-genome data to study a large, genetically diverse collection of historical and modern pneumococci, with the aim to better understand the mechanisms and role of capsular switching in pneumococcal evolution.

## METHODS

### Strains and Genome Sequencing

A global collection of 426 pneumococci recovered during 1937–2007 (Supplementary Materials) were previously serotyped by the Quellung reaction and genotyped by MLST [23] to determine the sequence type (ST). Closely related isolates were assigned to clonal complexes (CCs) by a modified goeBURST method [29] (Supplementary Materials). CCs were named after the predicted founder ST. When no single founder ST could be determined, CCs were named NoneX, where X was the ST of lowest numeric value within the CC. Isolates of the same CC but with different serotypes were presumed to be capsular switch variants.

A total of 96 genetically diverse isolates from our collection were selected for whole-genome sequencing (Supplementary Materials and Table 1) on the Illumina platform as previously described [30]. Seven were excluded as technical failures. Raw sequence data were assembled using Velvet [31], and contigs were deposited in a BIGS database (BIGSdb) [32]. Sequence data were deposited in the European Nucleotide Archive (Supplementary Table 1).

### Nucleotide Sequence Analysis

Eight CCs were represented by isolates subjected to whole-genome sequencing and were selected for further *cps* locus analysis; 7 CCs included capsular switch variants. Within each CC, the ancestral serotype was assumed to be the most common one or that of the oldest isolate. *cps* sequence alignments for study isolates and published loci were used to investigate serotype changes. Nucleotide sequences of the *cps* locus, and upstream/downstream flanking sequences where appropriate, were aligned using MUSCLE [33] and imported to MEGA5 [34] for visual inspection of variable sites. When recombination at the *cps* locus was predicted, potential donor representatives were sought from our whole-genome sequenced isolates, the *cps* locus reference sequences, and an additional 131 pneumococcal genomes retrieved from GenBank. Putative donors were identified as isolates for which the *cps* locus differed from that of the recombinant by a maximum of 3 nucleotides (excluding clusters of closely linked substitutions, which were considered to have arisen through recombination within the *cps* locus itself). MLST data were used to infer the CC most likely represented by the true donor isolate (the same as that of the donor representative). Recombination regions were identified as the minimum region over which the recombinant representative (hereafter, the “recombinant”) differed from the ancestral representative (hereafter, the “ancestor”) and was identical or highly similar (>99.7% sequence identity) to the donor representative (hereafter, the “donor”). Where no suitably matched donor was identified, the maximum recombination region was estimated as the maximum length over which the recombinant differed from the ancestor (Supplementary Materials).

## RESULTS

### Indication of Serotype Switching

**Figure 1.**
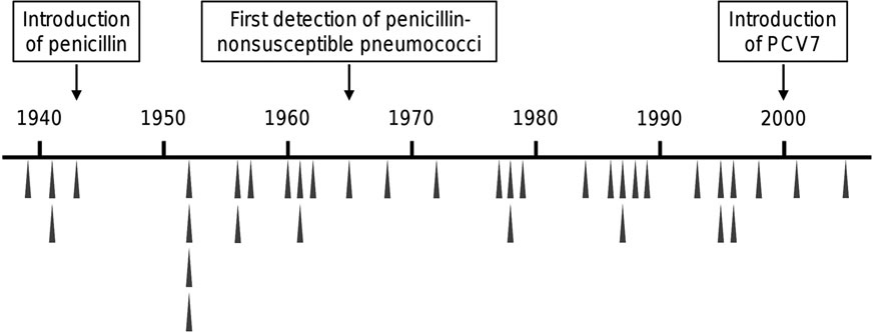
Temporal distribution of capsular switch variants among the historical isolate collection. Gray triangles indicate isolation dates for the oldest recombinant representatives of 36 independent capsular switch events identified among the historical pneumococcal collection. Abbreviation: PCV7, 7-valent pneumococcal conjugate vaccine.

Among the entire collection of 426 pneumococci, 21 of 163 unique CCs were represented by isolates of 2–6 serotypes. At least 36 independent changes of serotype within CCs were represented in our collection, 34 of which predated the introduction of pneumococcal conjugate vaccines (Figure 1).

### Within-CC *cps* Diversity

#### CC15

Seven of 10 CC15^14^ (CC15, serotype 14) *cps* locus sequences were unique (Table 1). Isolates ICE13 and ICE50 shared *wciY* locus nucleotide substitutions with 4 CC124^14^ representatives (isolates 14/9, USA6, Ala289, and ICE594), suggesting a possible recombination event between these CCs. The CC15^14^ representatives had a 432-bp deletion in the *wciY* gene, resulting in a predicted 250-amino-acid truncation of the protein. Isolate CGSP14 [35] also had an additional 52-bp deletion within the *wciY* gene; if this represented a true deletion event rather than a sequencing/assembly error, the resultant protein would be further reduced to 38 amino acids.

**Table 1.** Genetic Evolution at the *cps* Locus Among Isolates Representing the Same Pneumococcal Serotypes and Clonal Complexes (CCs)

CC^Serotype^	Isolate	Year	Nucleotide Substitution(s) as Compared to Ancestor (*gene*)	Recombination Regions
15^14^	14/5	1967	NA	…
	PMEN10	1987	A8695G (*wchM*); missing bp 11 509–11 525 and bp 12 423–end^a^	…
	SPnINV200	1995	C12439T (*wciY*)	…
	ICE13	1998	C12466A and C12467A (*wciY*)	…
	Ala243	1998	C12439T (*wciY*)	…
	GA13338	1999	C12439T (*wciY*)	…
	Ala317	2001	None	…
	ICE50	2003	C12439T, C12466A, C12467A, and G12502A (*wciY*); 22-bp insertion at position 11 509	…
	CGSP14	2004–2005	C1010T (*wzg*) and C12439T (*wciY*); missing bp 11 488–11 539	*cps* bp 1–367
	SP14-BS292	Unknown	T1976A (*wzh*) and C12439T (*wciY*)	…
66^9N^	9N/6	1960	NA	…
	USA8	2001	A4399G (*wchA*), A14000G, T14014G, C14037T, and T14042C (between *ugd* and IS1381 putative transposase)	…
113^18C^	18C/2	1939	NA	…
	18C/1^b^	1940	A2203G (*wzd*)	…
	18C/3	1968	G3478A (*wze*)	…
	Netherlands^18C^-36	1980	None	…
	USA2	1999	C4622T (*wchA*)	*cps* bp 15 861–17 005
	ICE501	2002	None	…
124^14^	14/2^b^	1952	NA	…
	14/4	1961	A3109G and G3478A (*wze*), A5616G and C5617T (*wchK)*, and T6662A (*wzy*)	*cps* bp 12 330–12 467
	PMEN35	1980	None; missing bp 12 437–end^a^	…
	14/9	1982	T11491C, A11524G, C12466A, C12467A, G12495A, and A12505T (*wciY*)	…
	14/7	1992	A4141G (*wchA*)	…
	Ala292	1998	C3816A and G4087A (*wchA*)	…
	USA6	1999	C43A (*wzg*), A12403G, C12466A, C12467A, and G12502A (*wciY*)	…
	Ala263	2002	None	…
	Ala289	2002	C12466A, C12467A, and G12502A (*wciY*)	…
	ICE46	2003	None	…
	ICE594	2005	C12466A, C12467A, and G12502A (*wciY*)	…
	CCR11974		G2455T (*wzd*)	…
156/162^9V^	9V/5	1991	NA	…
	PMEN3	1993	None	…
	GA08780	1997	T5230C (*wchO*)	…
	SP9-BS68	Unknown	None	…
191^7F^	7F/3	1962	NA; missing bp 20 430–end^a^	…
	7F/5^b^	1962	A16G (*wzg*)	…
	Netherlands^7F^-39	1984	None; missing bp 20 434–end^a^	…
	7F/4	1986	None; missing bp 20 434–end^a^	…
	ICE22	1993	C20435T, A20436G, T20441C, and A20450G (*glf*)	…
	CDC1087–00	1999	T10697C (*wchF*)	…
	USA16	2003	None	…
218^12F^	12F/5	1988	NA	…
	Denmark^12F^-34	1995	G16593T (between *fnlA* and *fnlB*)	…
	12F/6	1996	G16593T (between *fnlA* and *fnlB*) and C18121T (*fnlC*)	…
	USA18	1999	G16593T (between *fnlA* and *fnlB*)	…
	CDC0288-04	2003–2004	C7397T (*wzy*), G16593T (between *fnlA* and *fnlB*), and C18121T (*fnlC*)	…
218^7F^	7F/2	1952	NA	…
	ICE23	1993	C5369A (*wchF*), C6499T (*wcwA*), and A12622C (*wcwH*)	…
	USA20	1999	C9059T (*HG140*), C11766G (*wcwH*); missing bp 20 431–end^a^	…

Abbreviation: NA, not applicable.

^a^ Likely due to sequencing/assembly failure.

^b^ This isolate was used as the *cps* reference strain in reference [2].

#### CC66

**Figure 2.**
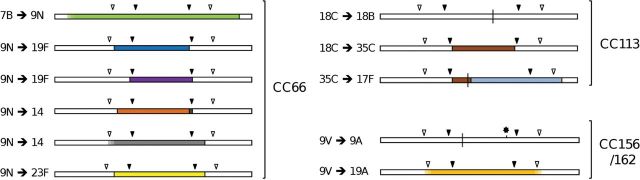
Genetic changes leading to capsular switching within clonal complexes (CCs) 66, 113, and 156/162. Bars represent the *cps* locus and flanking regions. Filled arrows indicate the relative positions of the *dexB* (left) and *aliA* (right) loci. Open arrows indicate the relative positions of the *pbp2x* (left) and *pbp1a* (right) loci. Vertical bisecting lines indicate single nucleotide substitutions. Recombination fragments are indicated by coloring. Identical sequences are indicated by identical coloring. Regions bounded by solid lines are depicted at their maximum length. Regions not bounded by solid lines are depicted at their minimum length (Table 2). The asterisk indicates a single-base-pair deletion. The schematic is drawn approximately to scale.

Ten CC66 isolates, dated 1952–2005, represented 5 serotypes. The oldest isolate was serotype 7B; the next oldest was a serotype 9N from 1960. An approximately 53.4-kb region of sequence of the serotype 9N isolate, including *pbp2x,* the *cps* locus, and *pbp1a*, differed from the CC66^7B^ isolate but was highly similar to a CC3782^9N^ isolate dated 1952 (Table 2 and Figure 2). (Although the data strongly suggest that a CC3782^9N^ pneumococcus was the donor in this case, it is possible that another clone not represented in this sample was the true donor. Thus, we use the term “CCX^X^-like” when describing putative donors.) Within the *cps* locus, a second CC66^9N^ isolate differed from the oldest CC66^9N^ isolate by only 5 nucleotide substitutions (Table 1).

**Table 2.** Genetic Changes Leading to Capsular Switching Events Among Pneumococci

					Recombination			
CC	Serotype Change	Year^a^	PM or R	Point Mutation(s)	Donor	Breakpoints	Import, kb	*cps* Point Mutations as Compared to Donor^c^
66	7B→9N	1960, 2001	R	…	CCNone3782^9N^	≥8901 bp 5’ *pbp2x*–7415 bp 3’ *pbp1a*	≥54.3	None^d^
	9N→19F	1972	R	…	Unk^19F^	2538 bp 5’ *dexB*–34 bp 5’ *aliA*	≤23.7	…
	9N→19F	2005	R	…	Unk^19F^	115 bp 3’ *dexB*–bp 1173 *aliA*	≤20.6	…
	9N→14	1995, 1999	R	…	CC15^14^	2158 bp 5’ *dexB*–between bp 88 *aliA* and 785 bp 3’ *aliA*	23.1–25.8	A8695G, G10976T, and C12439T
	9N→14	1997	R	…	CC15^14^	Und	Und	A4486G, A8695G, and G10461A^e^
	9N→14	unk^a^	R	…	CC124^14^	≥2520 bp 5’ *dexB*–4062 bp 3’ *aliA*	≥29.9	A1704G and T12439C
	9N→23F	2001	R	…	Unk^23F^	1964 bp 5’ *dexB*–3013 bp 3’ *aliA*	≤30.4	…
113	18C→18B	1941^b^	PM	G12382T	…	…	…	…
	18C→35C	1941, 1943^b^	R	…	Unk^35C^	144 bp 3’ *dexB*–76 bp 5’ *aliA*	≤19.0	…
	35C→17F	1939	R	…	CC574^17F^	bp 549 *wze*–3278 bp 3’ *pbp1a*	30.9	A8794G
	18C→9V	1968^b^	R	…	Unk^9V^	Und	Und	…
124	14→11C	1957^b^	R	…	Unk^11C^	Und	Und	…
	14→9L	1952	R	…	Unk^9L^	Und	Und	…
156/162	9V→9A	1962^b^	PM	G1543A, deletion bp 16 996	…	…	…	…
	9V→19A	2005, 2006	R	…	CC199^19A^	≥5701 bp 5’ *dexB* to ≥6022 bp 3’ *aliA*	≥ 50.4	None
191	7F→7A	1937^b^	PM	A2375G, A10521T, insertion bp 8606	…	…	…	…
218	12F→7F	1952, 1993, 1999	R	…	CC191^7F^	≥9045 bp 5’ *pbp2x*–4782 bp 3’ *pbp1a*	≥ 58.2	C6854A, C8089T, and 2 putative recombinations marked by 39 nucleotide changes bp 1083–1279 and 7 changes bp 19 486–20 473
574	17F^b^→2	1956^b^	R	…	CCNone128^2^	Between bp 326 *dexB* and 639 bp 3’ *dexB*–5229 bp 3’ *pbp1a*	34.9–36.8	G3294T, T3339C, and G3956T

Abbreviations: CC, clonal complex; PM, point mutation; R, recombination; Unk, unknown; Und, undetermined.

^a^ Year of recipient isolate(s) with the new serotype.

^b^ This isolate was used as the *cps* locus reference strain in reference [2].

^c^ Calculated over the region spanning the synthesis-related genes only (Supplementary Materials).

^d^ Missing bp 14 006–15 542 (end) likely due to sequencing/assembly failure.

^e^ Missing bp 12 437–12 516 (end) likely due to sequencing/assembly failure.

Sequences of the 2 CC66^19F^ genomes suggested 2 independent capsular switches, but no potential 19F donors were identified. The putative imports were estimated as the regions over which these genomes clearly differed from those of CC66^9N^; independent recombination breakpoints were indicated (Table 2 and Figure 2).

There were 3 independent capsular switches among 4 CC66^14^ representatives. On the basis of sequence similarity, the donors for 2 events were CC15^14^-like, while the third was CC124^14^-like. The breakpoints for 1 event could not be determined because of extensive diversity in the *cps* flanking regions, but breakpoints for the others were estimated (Table 2 and Figure 2). Interestingly, the 5′ breakpoints for 2 serotype 14 switches and one of the aforementioned serotype 19F switches were all 2.2–2.5 kb upstream of *dexB* (in the ATCC700669 reference genome [36], this corresponds to the *clpL* locus, which has been shown to be involved in modulating the expression of virulence-related genes [37] and in the development of penicillin nonsusceptibility [38]).

The single CC66^23F^ representative in our collection differed from the CC66^9N^ isolates from approximately 2 kb upstream of *dexB* to approximately 3 kb downstream of *aliA*, suggesting a potential recombination import of approximately 30.4 kb, but no suitable donor was identified.

#### CC113

Eleven CC113 isolates represented serotypes 18C (n = 6), 35C (n = 2), and 9V, 17F, and 18B (n = 1 each). Serotype 18C was assumed to be the ancestral serotype and was also represented by 1 of the 2 oldest isolates, both dated 1939. The change from serotype 18C to 18B was due to a single nucleotide substitution within the *wciX* gene [2], while each of the other serotype changes was associated with a recombination event. Both CC113^35C^ isolates likely arose from a single capsular switch event (Table 2 and Figure 2). The serotype 17F change appears to have involved DNA acquisition from a CC574^17F^-like pneumococcus by a CC113^35C^ pneumococcus. The 5′ region of the CC113^17F^ *cps* locus was distinct from that of 3 other serotype 17F isolates in our collection but differed by only 1 nucleotide substitution from the CC113^35C^ representatives. The CC113^17F^ genome was highly similar to that of a CC574^17F^ isolate between position 549 of the *wze* locus and approximately 3 kb downstream of *pbp1a*, after which the CC113^17F^ genome resembled that of the CC113^18C^ isolates.

**Figure 3.**
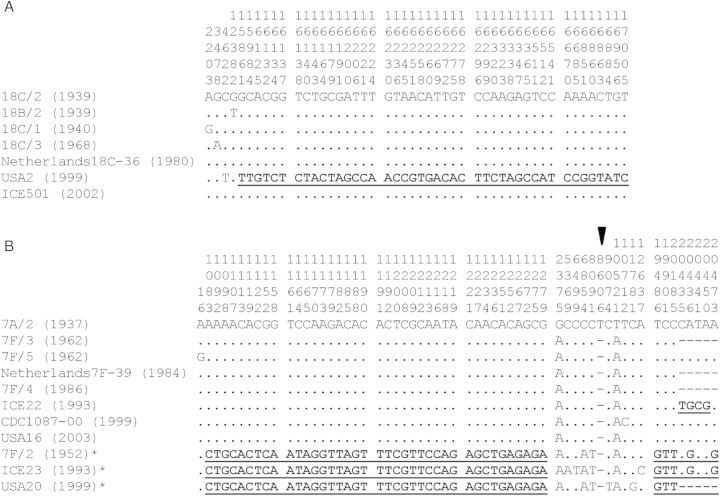
Variable site maps for the *cps* loci of serogroups 18 and 7. Rows represent the nucleotide sequences of independent isolates and are labeled as isolate name (year)*.* Nucleotide differences relative to row 1 are shown. Periods indicate an identical base, and hyphens indicate a missing base. Numbers above each column indicate the nucleotide position within the alignment. Nucleotide substitutions marking putative recombination regions are marked in bold and underlined. *A*, CC113 isolates include 1 of serotype 18B (18B/2) and 6 of serotype 18C. *B*, CC191 isolates include 1 of serotype 7A (7A/2) and 7 of serotype 7F. CC218 isolates all represent serotype 7F (B*). The arrow indicates the position of a single-base-pair insertion in the 7A/2 sequence, compared with the other serogroup 7 sequences.

There was little to no variation at the *cps* locus among 5 CC113^18C^ representatives (Table 1). A sixth isolate differed by 1 nucleotide in the *wchA* gene and by a putative import of approximately 1.1 kb (marked by 44 nucleotide substitutions) spanning the *rmlA* and *rmlC* genes (Figure 3*A*).

#### CC124

Twelve of 14 CC124 representatives were serotype 14 and represented 8 unique but highly similar *cps* sequences (Table 1). Two isolates were serotypes 9L and 11C, but no suitable donors were identified, and the *cps* flanking regions of both isolates were highly divergent from those of the CC124^14^ representatives for ≥30 kb in either direction. Thus, the putative recombination breakpoints could not be estimated.

#### CC156/162

Seven isolates (dated 1952–2006) representing serotypes 9V, 9A, and 19A were assigned to CC156/162. Four isolates were serotype 9V; the oldest was serotype 9A. The CC156/162^9A^ *cps* locus differed from that of the oldest CC156/162^9V^ locus by 2 nucleotides [2] (Table 2 and Figure 2). Three of the 4 CC156/162^9V^ *cps* loci were identical, and the other differed by 1 nucleotide.

Analysis of the *cps* loci and flanking regions of the 2 CC156/162^19A^ representatives (GenBank accession numbers AGOR01000001–AGOR01000024 and AGQA01000001–AGQA01000007) suggested a single capsular switch event. Approximately 50.4-kb regions of the CC156/162^19A^ genomes were highly similar to those of a CC199^19A^ isolate (Table 2 and Figure 2) and included *pbp2x,* the *cps* locus, and *pbp1a*. The regions directly flanking the putative import did not resemble those of the CC156/162^9V^ representatives, and thus the true recombination breakpoints could not be estimated. However, we deduced that the original import was ≥50.4 kb.

#### CC191

**Figure 4.**
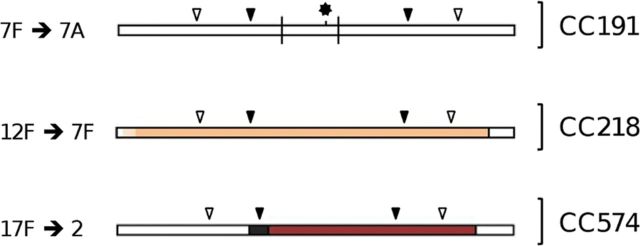
Genetic changes leading to capsular switching within clonal complexes (CCs) 191, 218, and 574. Bars represent the *cps* locus and flanking regions. Filled arrows indicate the relative positions of the *dexB* (left) and *aliA* (right) loci. Open arrows indicate the relative positions of the *pbp2x* (left) and *pbp1a* (right) loci. Vertical bisecting lines indicate single nucleotide substitutions. Recombination fragments are indicated by coloring. Regions bounded by solid lines are depicted at their maximum length. Regions not bounded by solid lines are represented at their minimum length (Table 2). The asterisk indicates a single-base-pair insertion. The schematic is drawn approximately to scale.

Seven of the CC191 representatives were serotype 7F, and the eighth, and oldest, was serotype 7A. Serotypes 7A and 7F differed by 3 nucleotides (Table 2 and Figure 4). There was a repeated motif (5′-CTA AGA TGA ATA-3′) within the *wcwC* gene, and the number of repeats differed between isolates (n = 3, 4, or 6). Repeat motifs are difficult to sequence accurately, so further speculations about such changes cannot be made. Apart from the repeat region, the CC191^7F^ *cps* loci each differed by a maximum of 4 nucleotides (Table 1 and Figure 3*B*).

#### CC218

Eight CC218 isolates represented serotypes 12F (n = 5) and 7F (n = 3). Between approximately 9 kb upstream of *pbp2x* and approximately 4.8 kb downstream of *pbp1a*, CC218^7F^ and CC191^7F^ sequences were very similar, although differentiating nucleotides were identified (Table 2). The clustering of 44 nucleotide substitutions within the *cps* locus was indicative of recombination events (Figure 3*B*), but whether these events occurred before or after the *cps* switch is unknown. Note that approximately 585 bp were missing between *pbp2x* and *dexB* in both CC218^7F^ and CC191^7F^ representatives. Table 1 provides additional details about nucleotide substitutions among the *cps* loci of CC218^7F^ and CC218^12F^ isolates.

#### CC574

Two CC574 isolates were examined: a serotype 17F isolate from 1952 and a serotype 2 isolate from 1956. The sequences differed from position 326 of *dexB* to approximately 5 kb downstream of *pbp1a*. A region of the serotype 2 representative (totaling approximately 35 kb, from approximately 0.6 kb downstream of *dexB* to approximately 5 kb downstream of *pbp1a*) was highly similar to that of a CCNone128^2^ representative dated 1916 (Table 2 and Figure 4). A short, 314-bp region of unknown sequence in the *dexB* locus was also present.

## DISCUSSION

Within our collection of historical and modern pneumococci, we identified 36 independent capsular switching events. Approximately 94% of the variants were isolated prior to the introduction of PCV7 and were roughly evenly distributed through time (Figure 1). The collection was not designed for inferring a capsular switching rate; nevertheless, these data imply that this phenomenon has been a regular occurrence (ie, there is evidence of capsular switching within a diverse range of CCs) every decade throughout the past 7 decades).

Analysis of the *cps* loci of 10 representatives of CC66 indicated multiple independent changes to the same serotype, supporting the notion that capsular switching may occur regularly among pneumococci. Within this CC, an initial capsular switch from serotype 7B to 9N no later than 1952 was followed by at least 2 independent changes to serotype 19F no later than 1972 and 2005 and by at least 3 independent changes to serotype 14, each no later than the mid-1990s. This is consistent with previous studies that demonstrated multiple changes of serotype within the same CC: CC156/162^9V→14^ [39–41], CC81^23F→19F/A^ [30], and CC695^4→19A^ [25, 28].

**Figure 5.**
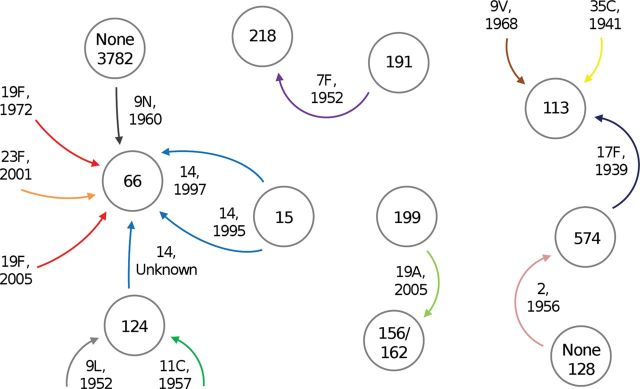
Inter-clonal complex (CC) *cps* locus transfers inferred in this study. Circles represent pneumococcal CCs as named. Arrows indicate transfer of *cps* loci between CCs, as inferred from nucleotide sequence analyses for 15 capsular switching events. Donor CCs could not be predicted for a total of 7 events because donor isolates were not present among this collection. Arrow labels indicate serotypes associated with the transferred *cps* loci and isolation year of the oldest recombinant pneumococcus in this collection.

We studied 18 capsular switching events in detail. Three of these were presumably the result of nucleotide substitution and/or deletion, a finding consistent with previous work [2]. The remaining 15 events appeared to be due to recombination, and breakpoints for 11 events could be estimated by comparison of the *cps* loci and flanking sequences of the putative ancestors, donors, and recombinants. The capsular switch recombination fragments identified here (ie, imports of various lengths, inserted at different points around the *cps* locus, with or without the adjacent *pbp* sequences) are consistent with fragments detected in previously published studies [25, 42, 43]. Figure 5 depicts the exchange of c*ps* loci between CCs, as inferred by our data. Isolates identified as donors from our collection could possibly have represented different CCs than the true donors; however, even if this were true, our conclusions about the number of independent capsular switches and the range of recombination fragment sizes would remain unchanged.

Our analyses did not indicate any recombination breakpoint hot spots around the *cps* locus, which had also not been indicated by any previous studies [25, 27, 28, 30]. It is impossible to know whether the putative imports were acquired through single or multiple recombination events, but the former is most parsimonious. The import lengths were estimated to range from approximately 19.0 to ≥58.2 kb (and apart from 1 example, always included the entire *cps* locus) and did not show any trends toward increasing/decreasing lengths through time.

The CC218^12F→7F^ and CC66^7B→9N^ events were characterized by large (>50 kb) recombination imports and must have taken place no later than 1952 and 1960, respectively. Large-scale recombination between pneumococci has therefore clearly been occurring for decades, although the technology capable of detecting and detailing such events has only recently become available [25, 28, 30, 42]. Additionally, both of these putative imports included the *pbp2x* + *cps* + *pbp1a* loci, as reported for other events [27, 28] and for another newly characterized event in this study (CC156/162^9V→19A^).

Recently reported in vivo *pbp2x* ± *cps* ± *pbp1a* recombination events were detected soon after widespread vaccination began in the United States. No such recombinants had been reported before in nature, and the imported *pbp2x* and *pbp1a* sequences conferred penicillin nonsusceptibility. Thus, it was theorized that vaccine-induced and/or antibiotic-induced selective pressures may play a role in driving these genetic changes [28, 44]. Penicillin was introduced in the 1940s; oral penicillins were not available until the mid-1950s, and their use was initially limited. Consequently, our analyses question the above theory because the CC218^12F→7F^ and CC66^7B→9N^ events were both associated with penicillin-susceptible pneumococci and occurred before the widespread use of penicillins and before the introduction of PCV7. Our new data suggest that recombination of the *cps* locus and flanking regions might be “normal” biological processes, the evolution of which has undoubtedly been influenced by naturally occurring immunity and other selective pressures. Presumably, vaccine-induced immune pressures and/or the pressure of antibiotic use subsequently influence the spread and maintenance of advantageous genes (and/or alleles) by selecting recombinants that are best able to survive. However, the potential negative effects should not be underestimated: the CC695^4→19A^ “vaccine escape” capsular switches in the United States were first recovered from patients with pediatric invasive pneumococcal disease only 3 years after PCV7 introduction and, 2 years later, were the third-most common serotype 19A CC causing invasive pneumococcal disease among all age groups [20]. These strains were penicillin nonsusceptible, owing to the simultaneous acquisition of altered *pbp2x* and *pbp1a* genes. Consequently, the increase in prevalence of these strains likely contributed to the increase in pneumococcal penicillin nonsusceptibility in the United States after PCV7 introduction [20, 45].

Another interesting finding was that a CC199^19A^-like representative was the most probable donor of the 19A *cps* locus and flanking *pbps* to the CC156/162^19A^ isolates described in this study. CC199^19A^ representatives were also the donors of the *cps* locus ± *pbps* to the vaccine escape progeny [25, 28] and an ST320^19→19A^ *cps* locus switch [27]. Future studies will attempt to uncover an explanation of why CC199^19A^ representatives appear to be “good” *cps* locus donors.

Our analyses also revealed recombination at the *cps* locus that did not result in capsular switching, among isolates belonging to the same ancestral lineages (CC113^18C^ and CC191^7F^) and different ancestral lineages (CC191^7F^ vs CC218^7F^ and CC15^14^ vs CC124^14^). Similar events have been noted within the CC81^23F^ lineage [26] and among serogroup 6 and 19 isolates [10–12]. We speculate that this is more likely to be the result of recombination whereby some genes are coincidentally exchanged, rather than the result of exchange that occurred directly in response to selection pressure. It could also be the result of DNA repair mediated by recombination at or near the *cps* locus. A comparison of the *cps* loci of CC15^14^ and CC124^14^ representatives also indicated a deletion in the CC15^14^ *wciY* gene, resulting in a predicted truncation of the putative glycerol phosphotransferase encoded by this gene. Notwithstanding the deletion, the CC15^14^ isolates were successfully serotyped by the Quellung reaction, indicating successful capsule production. Indeed, an in silico analysis of the predicted *cps* protein coding regions failed to identify a specific reaction catalyzed by the serotype 14 version of this protein [46].

Given the overall capacity for *cps* locus recombination, the associations between genotypes and serotypes are puzzling, as is our evidence of a high level of *cps* sequence conservation within some CCs (eg, CC191^7F^ and CC218^12F^). Perhaps there is some synergism between serotype and the genetic background of a strain that conveys an advantage to certain combinations over others. This sort of synergistic effect has been invoked to explain changes in the pneumococcal population in South Korea, where there was a prevaccine reduction of multidrug-resistant CC271/320^19F^ pneumococci and replacement by similarly multidrug-resistant CC271/320^19A^ pneumococci over several years [47]. It is also possible that genes, such as those encoding sugar-biosynthesis enzymes, outside the *cps* locus contribute to capsular expression and facilitate a genotype/serotype association [2].

Our collection of pneumococci provided a unique opportunity to study evolution at the *cps* locus over approximately 70 years. Capsular switching with/without simultaneous *pbp* transfer has occurred regularly and prior to both PCV7 introduction and widespread antibiotic use. It is highly likely that the proliferation of newly generated NVT capsular switch variants will continue to be favored by PHiD-CV and PCV13 vaccination programs, as was the case after PCV7 implementation. Penicillin-nonsusceptible variants will have an even greater advantage. Although the magnitude of these selective forces relative to those favoring established genotype/VT associations remains unclear, the implementation of vaccine programs across the globe will most likely favor the intercontinental spread of NVT and penicillin-nonsusceptible pneumococci.

## Supplementary Data

Supplementary materials are available at *The Journal of Infectious Diseases* online (http://jid.oxfordjournals.org/). Supplementary materials consist of data provided by the author that are published to benefit the reader. The posted materials are not copyedited. The contents of all supplementary data are the sole responsibility of the authors. Questions or messages regarding errors should be addressed to the author.

Supplementary Data
