# Supplementary material for: Pneumococcal Capsular Switching: A Historical Perspective
Source: J Infect Dis. 2012 Nov 21;207(3):439–49. doi: 10.1093/infdis/jis703 (PMC3537446; doi:10.1093/infdis/jis703)
Supplement: Supplementary Data [file supp_207_3_439__index.html]

Pneumococcal Capsular Switching: An Historical Perspective — Pneumococcal Capsular Switching: A Historical Perspective — Pneumococcal Capsular Switching: A Historical Perspective — Supplementary Data 

# Pneumococcal Capsular Switching: A Historical Perspective

## Supplementary Data

Supplementary Data

**Files in this Data Supplement:**

- Supplementary Data - Docx file
