# Supplementary material for: Pneumococcal Capsular Switching: A Historical Perspective
Source: J Infect Dis. 2012 Nov 21;207(3):439–49. doi: 10.1093/infdis/jis703 (PMC3537446; doi:10.1093/infdis/jis703)
Supplement: Supplementary Data [file supp_jis703_jis703supp.docx]

**Supplementary information for: Pneumococcal Capsular Switching: An Historical Perspective**

Kelly L. Wyres^1^, Lotte M. Lambertsen^2^, Nicholas J. Croucher^3^, Lesley McGee^4^, Anne von Gottberg^5^, Josefina Liñares^6^, Michael R. Jacobs^7^, Karl G. Kristinsson^8^, Bernard W. Beall^4^, Keith P. Klugman^5,9^, Julian Parkhill^3^, Regine Hakenbeck^10^, Stephen D. Bentley^3^, Angela B. Brueggemann^1*^

^1^Department of Zoology, University of Oxford, Oxford, United Kingdom, ^2^Department of Microbiology Surveillance and Research, Statens Serum Institut, Copenhagen, Denmark, ^3^Pathogen Genomics Team, Wellcome Trust Sanger Institute, Hinxton, United Kingdom, ^4^ Streptococcus Laboratory, Centers for Disease Control and Prevention, Atlanta, Georgia, USA, ^5^Centre for Respiratory Diseases and Meningitis, National Institute for Communicable Diseases, Gauteng, South Africa, ^6^Hospital Universitari de Bellvitge, IDIBELL, CIBERes, Barcelona, Spain, ^7^Department of Pathology, Case Western Reserve University, Cleveland, Ohio, USA, ^8^Clinical Microbiology Department, Landspitali University Hospital and University of Iceland, Reykjavik, Iceland, ^9^Hubert Department of Global Health, Emory University, Atlanta, Georgia, USA, ^10^Department of Microbiology, University Kaiserslautern, Kaiserslautern, Germany.

**Introduction**

Twelve studies have documented pre-vaccine temporal changes in relative serotype prevalence [[1-13](#_ENREF_1)]. Only three [[4](#_ENREF_4), [7](#_ENREF_7), [9](#_ENREF_9)] included pneumococci isolated earlier than 1969; only two studies [[3](#_ENREF_3), [8](#_ENREF_8)] provided any genotype data.

**Methods**

*Historical pneumococcal collection*

The collection included 211 global pneumococci (dated 1937 – 1996) collected by the Statens Serum Institut; 88 of which were *cps* loci reference isolates, for which the full *cps* locus nucleotide sequence is available [[14](#_ENREF_14)]. 203 isolates were penicillin-susceptible. Also included were the 43 Pneumococcal Molecular Epidemiology Network reference clones (http://www.sph.emory.edu/PMEN/), plus 47 previously-characterised isolates from Iceland and the USA, Brazil, Germany, Poland and South Africa (dated 1990 - 2000s; 38 were penicillin-susceptible), that were closely related to STs represented by numerous older pneumococci. A literature search was performed to identify reports of early penicillin-nonsusceptible isolates. Available and viable isolates were added: 116 penicillin-nonsusceptible pneumococci and 9 penicillin-susceptible pneumococci dated 1969-1994 from Germany, Papua New Guinea, South Africa, Spain and the USA.

*Clonal complex (CC) assignment*

MLST data for all of our isolates and those deposited in the pneumococcal MLST database (www.mlst.net) were analysed by the goeBURST [[15](#_ENREF_15)] method to predict CC group and sub-group founders. Isolates were assigned to CCs so that only single/double locus variants of the group founder and any additional single locus variants of large (n ≥ 5 STs) sub-group founders were included. CCs were named according to the predicted group founder(s) ST(s). When goeBURST could not distinguish a group founder the group was designated NoneX, where X was the ST of lowest numerical value in the group. When isolates could not be assigned to a CC due to a lack of closely related STs, they were designated SingletonX, where X was the isolate ST.

*Selection of the ancestral serotype within each CC*

Within each CC the ancestral serotype was assumed to be that most commonly associated with members of the CC, or that represented by the oldest isolate. Change of serotype events resulting from point mutation(s) were differentiated from those resulting from recombination events by comparison of the published cps locus structures. When there was discordance between the serotype of the oldest representative and the most commonly represented serotype (n = 4 CCs), the ancestral type was inferred following further study. In two cases (CC156/162 serotype 9V and 9A, CC191 serotype 7F and 7A) the differences between the *cps* loci in question included nucleotide insertion/deletion whereby deletion events were associated with frame-shift mutations. Consequently the ancestral serotypes were assumed to be those which did not contain the deletion (i.e. those that retained a full-length coding sequence). Where the structure of the *cps* loci in question indicated that a change from one to the other was the result of a recombination event, we sought to infer the ancestral serotype by comparison of the *cps* locus and flanking genomic regions from these isolates and other representatives of the same serotypes but different CCs. There were two such cases (CC66 serotype 7B and 9N, CC218 serotype 12F and 7F), in both of which a candidate *cps* donor representative for one of the serotypes could be identified from our genome collection. Consequently it was possible to infer which of the two serotypes in question were most likely to represent a recombinant (i.e. not the ancestral type), because the *cps* locus and flanking genomic regions were highly similar to those of an isolate representing a different CC, plus at least one of the adjacent genomic regions were highly similar to that of isolates representing the alternative serotype in the same CC (the ancestral type). In these cases, the combination of ancestor/recombinant assignments that would explain the observed nucleotide sequence patterns by the simplest combination of recombination events (i.e. a single event) was assumed to be the most likely.

*Selection of strains for whole-genome sequencing*

96 isolates were selected for whole-genome sequencing (Table S1) based upon *pbp* sequence diversity (data not shown and currently under consideration elsewhere) and selection of CCs of interest (e.g. major international CCs) for which both historical and modern isolate representatives were available. Importantly, inclusion of the historical representatives allowed us to differentiate identical/highly similar sequence imports obtained exogenously versus identical sequence due to common ancestry. The selected isolates were distributed through time, by geographic location, by serotype and by *pbp* allele combination, in order to capture the greatest possible amount of genetic diversity.

*Sequence analysis of capsular switching events*

Serotype changes presumed to be the result of recombination at the *cps* locus were studied in further detail by comparing *cps* locus nucleotide sequences to identify potential *cps* locus donor representatives among our whole-genome sequenced isolates, the *cps* locus reference sequences, and an additional 131 pneumococcal genomes retrieved from Genbank. Regions spanning the synthesis-related genes of the reference *cps* loci (see below) were BLASTed against the sequences within the BIGSdb. Matching nucleotide sequences were extracted and aligned using MUSCLE [[16](#_ENREF_16)] and imported to MEGA5 [[17](#_ENREF_17)] for visual comparison of variable sites. *cps* locus sequences which were highly similar to the capsular switch representative in question (the putative recombinant) and which had been assigned to a different CC were considered as putative donor representatives. The *cps* locus flanking regions for the putative ancestor, donor and recombinant representatives were then retrieved from the BIGSdb [[18](#_ENREF_18)] by BLASTing and extracting each of the *dexB* and *aliA* loci plus flanking sequences (within the BIGSdb the user can request extraction of a specified length of sequence flanking the given BLAST matches). Sequences were aligned by MUSCLE [[16](#_ENREF_16)] and imported to MEGA5 [[17](#_ENREF_17)]. Recombination regions were identified as the minimum region over which the recombinant representative differed from the ancestral representative *and* was identical or highly similar (>99.7% sequence identity) to the donor representative. Where no suitably matched donor representative could be identified, the maximum recombination region was estimated as the maximum length over which the recombinant representative differed from the ancestral representative. Given the nature of next-generation sequencing technologies it was not possible to obtain completely contiguous sequence over the entire putative import length for all isolates. Missing sequence regions estimated at >100 bp length are stated in the text (estimated by comparison to the ATCC700669 reference genome; Genbank accession NC_011900.1 [[19](#_ENREF_19)]). Additionally, the transposase genes, which flank the functional coding genes of most *cps* loci, are notoriously difficult to sequence/assemble and as such were partially or completely missing for numerous isolates.

*Within serotype cps nucleotide sequence diversity*

Serotype changes resulting from point mutation(s) and within serotype, within CC *cps* evolution were studied by BLASTing regions of the reference *cps* locus nucleotide sequences spanning from the first to the last synthesis-related gene (i.e. excluding any flanking transposases, Table S2) against the appropriate genomes within the BIGSdb. These genes included the 6 universally conserved genes (*wzg, wzh, wzd*, *wze, wzx* and *wzy*) and all of the serotype-specific genes. An exception to this rule was the serotype 14 *cps* for which the 3’-most serotype-specific gene, *lrp*, contains a set of repeat regions which do not sequence/assemble well. Consequently, the *lrp* gene was excluded from the serotype 14 *cps* analyses. The *cps* locus sequences were extracted from the BIGSdb, aligned by MUSCLE and imported to MEGA5. Variable sites were identified and their positions within the alignment were mapped to coding loci using the Genbank reference annotations. Single bp insertions or deletions within poly-A/T sequence regions were assumed to result from sequence/assembly errors and were not subjected to further study. Isolates for which >2 such insertions or deletions were identified were excluded from the analyses, as were isolates for which the *cps* locus sequence was split across >2 assembly contigs.

**Results**

*Serotype 7F repeat motifs.*

As described in the main text, there was a repeated motif (5’-CTA AGA TGA ATA-3’) within the *wcwC* gene of the CC191^7A/7F^ *cps* loci. Three, four and 6 copies of the motif were present within three, two and three isolates, respectively (the latter three including the serotype 7A representative). It should be noted that the two serotype 7F loci which each contained 6 copies of the motif were each split across two assembly contigs. In both cases the first contig ended two bp short of the end of the third motif copy. Among CC218^7F^ representatives there were also differences in the number of copies of the *wcwC* repeat motif; two CC218^7F^ representatives each had a total of four copies of this motif and the third isolate had a total of six copies.

**References**

1. Barry MA, Craven DE, Finland M. Serotypes of *Streptococcus pneumoniae* isolated from blood cultures at Boston City Hospital between 1979 and 1982. J Infect Dis **1984**; 149:449-52.

2. Butler JC, Breiman RF, Lipman HB, Hofmann J, Facklam RR. Serotype distribution of *Streptococcus pneumoniae* infections among preschool children in the United States, 1978-1994: Implications for development of a conjugate vaccine. J Infect Dis **1995**; 171:885-9.

3. Choi EH, Kim SH, Eun BW, et al. *Streptococcus pneumoniae* serotype 19A in children, South Korea. Emerg Infect Dis **2008**; 14:275-81.

4. Feikin DR, Klugman KP. Historical changes in pneumococcal serogroup distribution: Implications for the era of pneumococcal conjugate vaccines. Clin Infect Dis **2002**; 35:547-55.

5. Fenoll A, Granizo JJ, Aguilar L, et al. Temporal trends of invasive *Streptococcus pneumoniae* serotypes and antimicrobial resistance patterns in Spain from 1979 to 2007. J Clin Microbiol **2007**; 47:1012-20.

6. Fenoll A, Jado I, Vicioso D, Pérez A, Casal J. Evolution of *Streptococcus pneumoniae* serotypes and antibiotic resistance in Spain: Update (1990 to 1996). J Clin Microbiol **1998**; 36:3447-54.

7. Finland M, Barnes MW. Changes in occurence of capsular serotypes of *Streptococcus pneumoniae* at Boston City Hospital during selected years betwen 1935 and 1974. J Clin Microbiol **1977**; 5:154-66.

8. Foster D, Knox K, Walker AS, et al. Invasive pneumococcal disease: epidemiology in children and adults prior to implementation of the conjugate vaccine in the Oxfordshire region, England. J Med Microbiol **2008**; 57:480-7.

9. Harboe ZB, Benfield TL, Valentiner-Branth P, et al. Temporal trends in invasive pneumococcal disease and pneumococcal serotypes over 7 decades. Clin Infect Dis **2010**; 50:329-37.

10. Kyaw MH, Clarke S, Edwards GFS, Jones IG, Campbell H. Serotypes/groups distribution and antimicrobial resistance of invasive pneumococcal isolates: implications for vaccine strategies. Epidemiol Infect **2000**; 125:561-72.

11. Lagos R, Muñoz A, San Martin O, et al. Age- and serotype-specific pediatric invasive pneumococcal disease: insights from systematic surveillance in Santiago, Chile, 1994-2007. J Infect Dis **2008**; 198:1809-17.

12. Mufson MA, Stanek RJ. Bacteremic pneumococcal pneumonia in one American city: a 20-year longitudinal study, 1978-1997. Am J Med **1999**; 107:34S-43S.

13. Scheifele D, Halperin S, Pelletier L, Talbot J, Members of the Canadian Paediatric Society/Laboratory Centre for Disease Control Immunization Monitoring Program. Invasive pneumococcal infections in Canadian children, 1991-1998: Implications for new vaccination strategies. Clin Infect Dis **2000**; 31:58-64.

14. Bentley SD, Aanensen DM, Mavroidi A, et al. Genetic analysis of the capsular biosynthetic locus from all 90 pneumococcal serotypes. PLoS Genet **2006**; 2:262-9.

15. Francisco AP, Bugalho M, Ramirez M, Carriço JA. Global optimal eBURST analysis of multilocus typing data using a graphic matroid approach. BMC Bioinformatics **2009**; 10:152.

16. Edgar RC. MUSCLE: multiple sequence alignment with high accuracy and high throughput. Nucleic Acids Res **2004**; 32:1792-7.

17. Tamura K, Peterson D, Peterson N, Stecher G, Nei M, Kumar S. MEGA5: Molecular Evolutionary Genetics Analysis using maximum likelihood, evolutionary distance, and maximum parsimony methods. Mol Biol Evol **2011**; 28:2731-9.

18. Jolley KA, Maiden MC. BIGSdb: Scalable analysis of bacterial genome variation at the population level. BMC Bioinformatics **2010**; 10:595.

19. Croucher NJ, Walker D, Romero P, et al. Role of conjugative elements in the evolution of the multidrug-resistant pandemic clone *Streptococcus pneumoniae*^Spain23F^ ST81. J Bacteriol **2009**; 191:1480-9.

**Table S1. Isolates selected for whole-genome sequencing.**

| **Isolate^a^** | **Serotype** | **Year** | **Country** | **ST** | **CC** | **Penicillin Susceptibility^b^** |
| --- | --- | --- | --- | --- | --- | --- |
| PMEN9 | 14 | 1993 | England | 9 | 15 | S |
| ICE13 | 14 | 1998 | Iceland | 9 | 15 | S |
| ICE570 | 14 | 2002 | Iceland | 9 | 15 | S |
| Ala243 | 14 | 1998 | USA | 13 | 15 | I |
| Ala317 | 14 | 2001 | USA | 13 | 15 | I |
| ICE50 | 14 | 2003 | Iceland | 13 | 15 | I |
| 14/5 | 14 | 1967 | Denmark | 15 | 15 | S |
| PMEN10 | 14 | 1987 | CSR | 20 | 15 | R |
| PMEN5 | 14 | 1990 | Spain | 18 | 18 | I |
| ICE57^c^ | 9N | 1994 | Iceland | 66 | 66 | S |
| USA9 | 14 | 1999 | Brazil | 66 | 66 | I |
| USA12 | 23F | 2001 | Poland | 66 | 66 | S |
| USA13 | 19F | 2005 | Germany | 66 | 66 | S |
| USA11 | 14 | Unknown | Brazil | 66 | 66 | S |
| PMEN18 | 14 | 1997 | USA | 67 | 66 | R |
| 9N/6 | 9N | 1960 | Denmark | 71 | 66 | S |
| 19F/11 | 19F | 1972 | Denmark | 71 | 66 | S |
| 7B/2 | 7B | 1952 | USA | 7180 | 66 | S |
| USA8 | 9N | 2001 | USA | 8119 | 66 | S |
| ICE211^c^ | 23F | 1999 | Iceland | 81 | 81 | I |
| PMEN2 | 6B | 1988 | Spain | 90 | 90 | I |
| PMEN12 | 6B | 1987 | Finland | 238 | 90 | R |
| PMEN22 | 6B | 1995 | Greece | 273 | 90 | S |
| PMEN36 | 18C | 1980 | Netherlands | 113 | 113 | S |
| ICE186^c^ | 18C | 1996 | Iceland | 113 | 113 | S |
| USA2 | 18C | 1999 | USA | 113 | 113 | S |
| ICE501 | 18C | 2002 | Iceland | 113 | 113 | S |
| ICE269^c^ | 18C | 2005 | Iceland | 113 | 113 | S |
| 17F/2 | 17F | 1939 | Denmark | 123 | 113 | S |
| 9V/4 | 9V | 1968 | Denmark | 123 | 113 | S |
| 18C/2 | 18C | 1939 | Denmark | 4706 | 113 | S |
| 18B/2 | 18B | 1941 | Denmark | 4706 | 113 | S |
| 35C/3 | 35C | 1943 | Denmark | 5989 | 113 | S |
| 18C/3 | 18C | 1968 | Denmark | 7195 | 113 | S |
| 35C/2 | 35C | 1941 | Denmark | 7196 | 113 | S |
| 14/2 | 14 | 1952 | USA | 124 | 124 | S |
| PMEN35 | 14 | 1980 | Netherlands | 124 | 124 | S |
| 14/9 | 14 | 1982 | Denmark | 124 | 124 | S |
| Ala292 | 14 | 1998 | USA | 124 | 124 | S |
| USA6 | 14 | 1999 | USA | 124 | 124 | S |
| Ala263 | 14 | 2002 | USA | 124 | 124 | S |
| Ala289 | 14 | 2002 | USA | 124 | 124 | S |
| ICE46 | 14 | 2003 | Iceland | 124 | 124 | S |
| ICE594 | 14 | 2005 | Iceland | 124 | 124 | S |
| 14/4 | 14 | 1961 | Denmark | 134 | 124 | S |
| 9L/2 | 9L | 1952 | USA | 5979 | 124 | S |
| 14/7 | 14 | 1992 | Denmark | 7198 | 124 | S |
| 11C/1 | 11C | 1957 | USA | 7201 | 124 | S |
| 7A/2 | 7A | 1937 | Denmark | 191 | 191 | S |
| 7F/3 | 7F | 1962 | Denmark | 191 | 191 | S |
| PMEN39 | 7F | 1984 | Netherlands | 191 | 191 | S |
| ICE22 | 7F | 1993 | Iceland | 191 | 191 | S |
| ICE7^c^ | 7F | 1997 | Iceland | 191 | 191 | S |
| USA16 | 7F | 2003 | Brazil | 191 | 191 | S |
| 7F/4 | 7F | 1986 | Scotland | 7208 | 191 | S |
| 12F/5 | 12F | 1988 | Denmark | 218 | 218 | S |
| ICE23 | 7F | 1993 | Iceland | 218 | 218 | S |
| PMEN34 | 12F | 1995 | Canada | 218 | 218 | S |
| 12F/6 | 12F | 1996 | Denmark | 218 | 218 | S |
| USA18 | 12F | 1999 | USA | 218 | 218 | S |
| USA20 | 7F | 1999 | South Africa | 218 | 218 | S |
| 7F/2 | 7F | 1952 | USA | 7210 | 218 | S |
| PMEN15 | 23F | 1997 | Taiwan | 242 | 242 | I |
| PMEN17 | 6B | 1997 | USA | 384 | 385 | I |
| 17F/3 | 17F | 1962 | Denmark | 392 | 392 | S |
| 17F/4 | 17F | 1962 | Denmark | 392 | 392 | S |
| PMEN4 | 23F | 1991 | USA | 37 | 439 | I |
| 23A/2 | 23A | 1945 | Denmark | 439 | 439 | S |
| 23F/5 | 23F | 1979 | Germany | 439 | 439 | S |
| 23F/10 | 23F | 1996 | Denmark | 515 | 439 | S |
| ICE11 | 6B | 1998 | Iceland | 490 | 490 | S |
| USA22 | 6C | 2005 | USA | 490 | 490 | I |
| 2/3 | 2 | 1943 | Denmark | 3744 | 490 | S |
| 22A/2 | 22A | 1939 | Denmark | 7181 | 490 | S |
| 18F/1 | 18F | 1961 | USA | 7182 | 490 | S |
| 10F/2 | 10F | 1956 | USA | 7186 | 490 | S |
| 14/8 | 14 | 1976 | Denmark | 7206 | 554 | I |
| 17F/1 | 17F | 1952 | USA | 574 | 574 | S |
| 2/2 | 2 | 1956 | USA | 574 | 574 | S |
| 14/3 | 14 | 1939 | Denmark | 875 | 1106 | S |
| 12F/3 | 12F | 1961 | Denmark | 7228 | 4399 | S |
| 19F/8 | 19F | 1952 | Denmark | 7229 | 4399 | S |
| 19F/5 | 19F | 1962 | Denmark | 7229 | 4399 | S |
| PMEN3 | 9V | 1993 | France | 156 | 156/162 | I |
| 9V/5 | 9V | 1991 | Denmark | 162 | 156/162 | S |
| 9V/6 | 9V | 1994 | Denmark | 162 | 156/162 | S |
| 9A/1 | 9A | 1962 | USA | 312 | 156/162 | S |
| PMEN14 | 19F | 1997 | Taiwan | 236 | 271/320 | R |
| ICE27 | 19F | 1995 | Iceland | 655 | 422/476 | S |
| 19F/12^c^ | 19F | 1995 | Denmark | 87 | 87/88 | I |
| 9N/2 | 9N | 1952 | USA | 7205 | None3782 | S |
| PMEN13 | 19A | 1988 | South Africa | 41 | None41 | I |
| 23F/4 | 23F | 1967 | Australia | 7184 | Singleton7184 | I |
| 12F/2 | 12F | 1962 | USA | 7194 | Singleton7194 | S |
| 19F/7^c^ | 19F | 1952 | Denmark | 7230 | Singleton7230 | S |
| 19F/10 | 19F | 1963 | Denmark | 7230 | Singleton7230 | S |

1. PMEN = Pneumococcal Molecular Epidemiology Network (reference strain).
2. S = Susceptible, penicillin minimum inhibitory concentration (MIC) ≤ 0.06 µg/ml; I = Intermediate, MIC 0.12 - 1 µg/ml; R = Resistant, MIC ≥ 2.0 µg/ml.
3. Excluded from analyses for technical reasons.

**Table S2. *cps* loci included in this study.**

| **Serotype** | ***cps* Reference Length (bp)** | **Length Included in Analyses (bp)** | **Genes Included in Analyses (5' - 3')** |
| --- | --- | --- | --- |
| 2 | 20420 | 17500 | *wzg, wzh, wzd, wze, wchA, wchF, wchG, wchH, wzy, wchI, wzx, ugd, glf, rmlA, rmlC, rmlB, rmlD* |
| 7A | 23837 | 20475 | *wzg, wzh, wzd, wze, wchA, wchF, wcwA, wcwC, wcwD, HG140, wcwF, wcwG, wcwH, wzy, wzx, rmlA, rmlC, rmlB, rmlD, glf* |
| 7B | 20947 | 19545 | *wzg, wzh, wzd, wze, wchA, wchF, wcwI, wcwL, wcwK, wcxU, wzy, rbsF, wzx, rmlA, rmlC, rmlB, rmlD, glf* |
| 7F | 23811 | ~20450^a^ | *wzg, wzh, wzd, wze, wchA, wchF, wcwA, wcwC, wcwD, HG140, wcwF, wcwG, wcwH, wzy, wzx, rmlA, rmlC, rmlB, rmlD, glf* |
| 9A | 20356 | 17308 | *wzg, wzh, wzd, wze, wchA, wchO, wcjA, mnaA, wzy, wcjB, wzx, wcjC, wcjD, tnp, ugd, wcjE* |
| 9L | 17433 | 15541 | *wzg, wzh, wzd, wze, wchA, wchO, wcjA, mnaA, wzy, wcjB, wzx, wcjC, ugd, tnp, wcjE* |
| 9N | 17434 | 15542 | *wzg, wzh, wzd, wze, wchA, wchO, wcjA, mnaA, wzy, wcjB, wzx, wcjC, ugd, tnp, wcjE* |
| 9V | 20671 | 17309 | *wzg, wzh, wzd, wze, wchA, wchO, wcjA, mnaA, wzy, wcjB, wzx, wcjC, wcjD, tnp, ugd, wcjE* |
| 11C | 18006 | 13383 | *wzg, wzh, wzd, wze, wchA, wchJ, wchK, wcyK, wcwR, wcrL, wzy, wcwT, wcwU, wzx, gct* |
| 12F | 23596 | 19081 | *wzg, wzh, wzd, wze, wciI, wciJ, wcxB, wzy, wcxD, wcxE, wcxF, wzx, mnaB, mnaA, fnlA, fnlB, fnlC* |
| 14 | 19736 | 12516 | *wzg, wzh, wzd, wze, wchA, wchJ, wchK, wzy, wchL, wchM, wchN, wzx, wciY* |
| 17F | 22714 | 19846 | *wzg, wzh, wzd, wze, wchA, wchF, wcxG, abp1, abp2, wciP, wcrT, wcrU, wzy, wcrV, wzx, rmlA, rmlC, rmlB, rmlD, glf* |
| 18B | 21637 | 20252 | *wzg, wzh, wzd, wze, wchA, wchF, wciU, wciV, wciW, wzx, wzy, wciX, wciY, gct, HG94, rmlA, rmlC, rmlB, rmlD, glf* |
| 18C | 21637 | 20252 | *wzg, wzh, wzd, wze, wchA, wchF, wciU, wciV, wciW, wzx, wzy, wciX, wciY, gct, HG94, rmlA, rmlC, rmlB, rmlD, glf* |
| 19A | 18121 | 14949 | *wzg, wzh, wzd, wze, wchA, wchO, wchP, wchQ, wzy, wzx, mnaA, rmlA, rmlC, rmlB, rmlD* |
| 19F | 19616 | 14820 | *wzg, wzh, wzd, wze, wchA, wchO, wchP, wchQ, wzy, wzx, mnaA, rmlA, rmlC, rmlB, rmlD* |
| 23F | 21813 | 18653 | *wzg, wzh, wzd, wze, wchA, wchF, wzy, wchV, wchW, wzx, wchX, gtp1, gtp2, gtp3, rmlA, rmlC, rmlB, rmlD* |
| 35C | 19218 | 17648 | *wzg, wzh, wzd, wze, wchA, wciB, wzy, wcrI, wcrJ, wcrK, mnp1, wcrH, mnp2, wzx, wciG, glf, wcjE* |

1. See text for details.
